# Predictive biomarkers of survival in patients with advanced hepatocellular carcinoma receiving atezolizumab plus bevacizumab treatment

**DOI:** 10.1002/cam4.5161

**Published:** 2022-08-23

**Authors:** Young Eun Chon, Jaekyung Cheon, Hyeyeong Kim, Beodeul Kang, Yeonjung Ha, Do young Kim, Seong Gyu Hwang, Hong Jae Chon, Beom Kyung Kim

**Affiliations:** ^1^ Department of Gastroenterology, CHA Bundang Medical Center CHA University Seongnam Republic of Korea; ^2^ Department of Medical oncology CHA Bundang Medical Center, CHA University Seongnam Republic of Korea; ^3^ Department of Internal Medicine Ulsan University Hospital, University of Ulsan College of Medicine Ulsan Republic of Korea; ^4^ Department of Internal Medicine Yonsei University College of Medicine Seoul Republic of Korea

**Keywords:** atezolizumab, bevacizumab, hepatocellular carcinoma, neutrophil to lymphocyte ratio, des‐gamma‐carboxy prothrombin

## Abstract

**Background:**

Since atezolizumab plus bevacizumab (ATE+BEV) regimen for patients with unresectable hepatocellular carcinoma (HCC) was released quite recently, real‐world data are lacking. We evaluated efficacy, safety, and predictive biomarkers for survival in patients receiving ATE+BEV.

**Methods:**

Between 2020 and 2021, HCC patients receiving ATE+BEV at academic teaching hospitals were recruited. Treatment response was assessed using the Response Evaluation Criteria in Solid Tumors (version 1.1.).

**Results:**

Among 121 patients enrolled, the median age was 63 years, with male predominance (82.6%). Complete response, partial response, stable disease, and progressive disease were identified in 2.5%, 26.4%, 54.5%, and 16.6%, respectively. Patients with alpha‐fetoprotein and des‐gamma‐carboxy prothrombin (DCP) response, defined as ≥30% and ≥50% decreases, respectively, at the first response evaluation relative to baseline, and those with neutrophil‐to‐lymphocyte ratio (NLR) <2.5, had significantly higher objective response rates (42.6% vs. 21.5%, 50.0% vs. 26.2%, and 39.0% vs. 19.4%, respectively; all *p* < 0.05). During follow‐up, the median overall survival (OS) was not reached, and the median progression‐free survival (PFS) was 5.7 months. Multivariable analyses showed that macrovascular invasion (adjusted hazard ratio [aHR] 2.541; *p* = 0.017), DCP ≥186 mAU/ml (aHR 5.102; *p* < 0.001), NLR ≥2.5 (aHR 3.584; *p* = 0.001), and an NLR decrease ≥10% at the first response (aHR 0.305; *p* = 0.002) were independent predictors of OS, and DCP ≥186 mAU (aHR 2.311; *p* = 0.002) and NLR ≥2.5 (aHR 1.938; *p =* 0.012) were independent predictors of PFS. Grade ≥3 treatment‐related adverse events (AEs) occurred in 33 (27.3%) patients.

**Conclusion:**

ATE+BEV showed favorable efficacy and safety. Baseline high DCP and NLR may be useful prognostic predictors for OS and PFS.

## 1 INTRODUCTION

Hepatocellular carcinoma (HCC) is currently a major health problem worldwide because it is one of the most common malignancies, as the leading cause of cancer‐related mortality.^1^ HCC, which has the highest age‐standardized incidence rates of any cancer in the Republic of Korea,^2^ is also increasing in the Western counties. Unfortunately, a considerable portion of HCC is still diagnosed as advanced stage HCC case, primarily because it is usually asymptomatic until it progresses to an advanced stage.

The combined regimen of atezolizumab plus bevacizumab (ATE+BEV) had been approved as a new 1st‐line regimen to treat unresectable HCC cases in 2020. Atezolizumab and bevacizumab exert immune‐modulatory effects by blocking PD‐1/PD‐L1pathways and inhibiting neovascularization by suppressing the action of vascular endothelial growth factor (VEGF). In the IMbrave 150 phase 3 clinical trial, using this combined regimen, both OS and PFS were substantially extended to 19.2 and 6.9 months, in comparison with sorafenib arm (13.4 and 4.3 months), respectively.^3^, ^4^ Hence, according to the 2022 update of Barcelona Clinic Liver Cancer (BCLC) strategy, ATE+ BEV has become the 1st line option for advanced HCC, instead of sorafenib.^5^ However, since this combination regimen has been released quite recently, real‐world data on its therapeutic efficacy and safety as well as the predictive biomarkers of survival in patients with a variety of clinical conditions are lacking.

In this multi‐center study, we assessed clinical efficacy and safety of ATE+BEV to treat advanced stage HCC cases in the real‐world practice, and then identified clinical biomarkers predictive of improved survival outcomes in those receiving ATE+BEV.

## 2 MATERIALS AND METHODS

### 2.1 Patient characteristics and follow‐up

From the present multi‐center, observational study, patients who were diagnosed as unresectable HCC, histologically or clinically according to HCC guidelines^5^, ^6^, ^7^, ^8^ and treated with ATE+BEV regimen in the three university Hospitals (CHA Bundang Medical Center, Severance Hospital, and Ulsan University Hospital) between May 2020 and April 2021, were screened for inclusion. Patients who did not finish the first cycle of ATE+BEV treatment or undergo the first tumor response assessment were excluded.

Not only age, sex, and performance status but also blood parameters such as white blood cell count, alpha‐fetoprotein (AFP), des‐gamma‐carboxy prothrombin (DCP), and the neutrophil‐to‐lymphocyte ratio (NLR) were investigated. As an etiology, hepatitis B virus (HBV) infection was defined as hepatitis B surface antigen seropositivity for more than 6 months. Hepatitis C virus (HCV) infection was defined as positive anti‐HCV seropositivity. Patients who consumed ≥140 g of alcohol per week in women or ≥210 g of alcohol per week in men were defined as alcohol drinkers. To assess hepatic functional reserve, the albumin‐bilirubin (ALBI) grade^9^ and Child‐Pugh class were used. Furthermore, tumor number, tumor, size, macrovascular invasion (MVI), extrahepatic lesions as well as Barcelona Clinic Liver Cancer (BCLC) staging were assessed.^5^ Previous treatments including resection, trans‐arterial chemoembolization, local ablation, radiation, and systemic therapy were investigated. Data were also collected on endoscopy procedures and the presence of varices.

The Institutional Review Board of three hospitals approved this study and the ethical guidelines of the 1975 Declaration of Helsinki were followed. Informed consent was waived since the present study had a retrospective design.

### 2.2 Baseline assessment and Treatment regimens and evaluation of tumor response

Every 3 weeks, 1200 mg of ATE plus 15 mg per kilogram of body weight of BEV was intravenously administered, and efficacy and safety were evaluated every 6–12 weeks. In case of grade 5 toxicity or progressive disease (PD), ATE+BEV was discontinued. Treatment response was evaluated through the Response Evaluation Criteria in Solid Tumors 1.1 (RECIST 1.1), based upon imaging modalities including computed tomography and/or magnetic resonance imaging^10^; complete response (CR), partial response (PR), or stable disease (SD), and PD. Objective response rate (ORR) was the proportion of patients achieving CR or PR, whereas disease control rate (DCR) was the proportion of patients achieving CR, PR, or SD.

Along with radiological response, we evaluated biological response using the changes in AFP and DCP on the basis upon two previous literatures.^11^, ^12^ As a cutoff of AFP decline from 20% to 50%, we explored the optimal cutoff associated with the ORR; AFP response was finally defined as AFP decrease ≥30% at the first response assessment compared with baseline. As a cutoff of DCP decline, DCP response was defined as DCP decrease ≥50% at the first response assessment compared with baseline.

The safety was measured using the Common Terminology Criteria for Adverse Events (version 5.0).

### 2.3 Statistical analysis

Data of the variables are expressed as mean ± standard deviation, median (interquartile range [IQR]), and number (%). The differences between continuous and categorical variables were assessed by Student's t‐tests (or the Mann–Whitney tests) and chi‐square tests (or Fisher exact tests), respectively. OS and PFS were defined as intervals from the initiation of ATE+BEV regimen to death or last follow‐up and from the initiation of ATE+BEV regimen to PD or death whichever happens first, respectively. Survival curves were generated by the Kaplan–Meier method and compared by the log‐rank test. The independent predictors affecting OS and PFS were assessed using multi‐variable Cox proportional hazards regression model. Statistical analysis was performed using SPSS statistics (version 25.0; IBM Crop.), and *p* value <0.05 was considered to be statistically significant.

## 3 RESULTS

### 3.1 Patient characteristics

After excluding 6 patients who did not complete the first cycle of ATE+BEV treatment (*n* = 1) or who did not undergo the first tumor response assessment (*n* = 5), 121 patients were finally analyzed (Table 1 **)**. The median age was 63 years, with male predominance of 82.6%. As an etiology of HCC, HBV‐related HCC was most common; 68.6%. Patients with BCLC stage C and B were 83.5% and 16.5%, respectively. Patients with liver cirrhosis at baseline were observed in 90%. The median maximal diameter of tumors was 4.8 cm and multiple intrahepatic tumors were observed in 64.5% of the study population. Macroscopic vascular invasion and extrahepatic metastasis were observed among 41.3% and 56.2% of the study population, respectively. The median AFP level was 96 ng/ml (IQR, 7–1964), and the median DCP level was 186 mAU/ml (IQR, 35–4084). The median NLR was 2.5.

**TABLE 1:** Baseline characteristics of the patients

Variables	All (*n* = 121)
Age, years	63 (57–71)
Male	100 (82.6)
ECOG PS	
0	36 (29.8)
1	81 (66.9)
2	4 (3.3)
Etiology	
HBV	83 (68.6)
HCV	7 (5.8)
Alcohol	19 (15.7)
Others	12 (9.9)
BCLC stage	
B	20 (16.5)
C	101 (83.5)
Child‐Pugh Class	
A	109 (90.0)
B	12 (10.0)
ALBI grade	
1	67 (55.4)
2	54 (44.6)
Number of intrahepatic tumors	
0	15 (12.4)
1	28 (23.1)
2	29 (24.0)
3	8 (6.6)
>3	41 (33.9)
Maximal size of intrahepatic tumor, cm	4.8 (1.8–9.4)
Extrahepatic metastasis	68 (56.2)
Macrovascular invasion	50 (41.3)
AFP, ng/ml	96 (7–1964)
DCP, mAU/ml	186 (35–4084)
Neutrophil to lymphocyte ratio	2.5 (1.8–4.0)
Platelet to lymphocyte ratio	122.9 (87.1–180.7)
Lymphocyte to monocyte ratio	1.5 (1.5–2.2)
Previous treatment	
Surgery	33 (27.3)
Transarterial therapy	67 (55.4)
Radioablation therapy	10 (8.3)
Radiation therapy	26 (21.5)
Presence of varices	37 (30.6)
Treated varices at baseline	22 (18.2)

*Note*: Variables were presented as n (%) or median (IQR).

Abbreviations: AFP, alpha‐fetoprotein; ALBI, albumin‐bilirubin; BCLC, Barcelona clinic liver cancer; DCP, des‐gamma‐carboxy prothrombin; ECOG PS, European Cooperative Oncology Group performance status; HBV, hepatitis B virus; HCV, hepatitis C virus.

### 3.2 Treatment responses

Table 2 demonstrates the treatment responses. CR and PR were achieved in three (2.5%), and 32 (26.4%) patients, respectively, which provides an ORR of 28.9%. The SD and PD were evaluated when the patient had the best response, which were 54.4% and 16.5%, respectively; the DCR was 83.3%.

**TABLE 2:** Treatment responses assessed with RECIST version 1.1

Responses	Rate
Complete response	3 (2.5)
Partial response	32 (26.4)
Stable disease	66 (54.4)
Progressive disease	20 (16.5)
Objective response rate	28.9
Disease control rate	83.3

*Note*: Data are presented as *n* (%) or %.

We assessed the association between radiological response and biomarker results. Patients with an AFP response (AFP decrease ≥30% at the first response assessment compared with baseline) had a significantly higher ORR than those without such a response (42.6% vs. 21.5%, respectively; *p* = 0.017). Likewise, patients with a DCP response (DCP decrease ≥50% at the first response assessment compared with baseline) at the first treatment response evaluation also had a significantly higher ORR than those without (50.0% vs. 26.2%, respectively; *p* = 0.032). Furthermore, patients with NLR <2.5 had a significantly higher ORR than those with an NLR ≥2.5 (39.0% vs. 19.4%, *p* = 0.017).

### 3.3 Survival outcomes and their predictors

**FIGURE 1:**
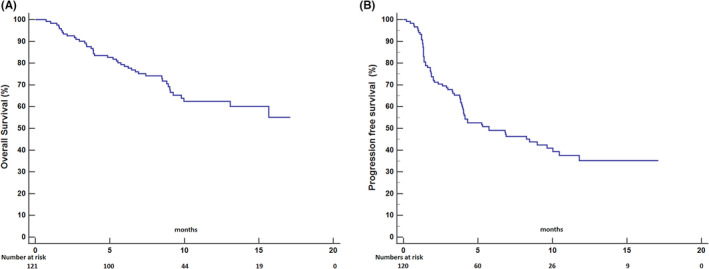
Kaplan–Meier plots of overall survival (A) and progression survival (B)

During the follow‐up (median 8.5 months; 95% confidence interval [CI] 6.6–12.3), a total of 42 (34.7%) patients died, while the median OS was not reached (Figure 1A). Seventy (57.9%) patients showed disease progression or died (median PFS, 5.7 months; 95% CI; 2.5–9.0) (Figure 1B).

Prognostic factors affecting OS and PFS are listed in Table 3. In univariable Cox regression analyses, ALBI grade, tumor number ≥2, tumor size ≥10 cm, MVI, DCP ≥186 mAU/ml, NLR ≥2.5, and an NLR decrease ≥10% at the first response were significant predictors of OS. In subsequent multivariable analyses, MVI, DCP ≥186 mAU/ml, NLR ≥2.5, and an NLR decrease ≥10% at the first response independently predicted OS (adjusted hazard ratios [aHRs], 2.541 [95% CI, 1.185–5.499; *p* = 0.017], 5.102 [95% CI, 2.118–12.287; *p* < 0.001], 3.584 [95% CI 1.661–7.733; *p* = 0.001], and 0.305 [95% CI, 0.144–0.643; *p* = 0.002], respectively). Likewise, univariable analyses showed that ALBI grade, tumor number ≥2, tumor size ≥10 cm, MVI, DCP ≥186 mAU/ml, and NLR ≥2.5 were significant predictors of PFS. Multivariable analyses identified DCP ≥186 mAU/ml, and NLR ≥2.5 as significant predictors of PFS (aHRs, 2.311 [95% CI 1.349–3.958; *p* = 0.002] and 1.938 [95% CI, 1.157–3.248; *p* = 0.012]), respectively. In contrast to radiological response, neither an early AFP response nor DCP response significant predicted OS or PFS. When we explored the other inflammatory markers in terms of platelet to lymphocyte ratio and lymphocyte to monocyte ratio, neither of them was a predictive for OS or PFS.

**TABLE 3:** Predictors for survival outcomes

	Overall survival			Progression free survival		
	Univariable	Multivariable		Univariable	Multivariable	
	*p*‐value	*p*‐value	Adjusted HR (95% CI)	*p*‐value	*p*‐value	Adjusted HR (95% CI)
Age, years, ≥63	0.975			0.341		
Male	0.508			0.428		
ECOG PS, 0/1 (vs. 2)	0.603			0.929		
Etiology, viral (vs. non‐viral)	0.914			0.264		
BCLC stage, C (vs. B)	0.336			0.785		
Platelet, /mm^3^, ≥150,000	0.053			0.216		
ALBI grade	<0.001	0.125	1.762 (0.855–3.634)	0.001	0.070	1.610 (0.962–2.695)
Tumor numbers, ≥2	0.049	0.143	1.927 (0.801–4.638)	0.008	0.119	1.601 (0.886–2.893)
Maximal tumor size, ≥10 cm	0.004	0.597	0.797 (0.344–1.845)	0.004	0.171	0.616 (0.308–1.232)
Extrahepatic metastasis	0.708			0.433		
Macrovascular invasion	0.001	0.017	2.541 (1.185–5.499)	0.046	0.245	1.366 (0.807–2.311)
AFP, ng/ml, ≥400	0.277			0.923		
DCP, mAU/ml, ≥186	<0.001	<0.001	5.102 (2.118–12.287)	<0.001	0.002	2.311 (1.349–3.958)
NLR, ≥2.5	<0.001	0.001	3.584 (1.661–7.733)	0.001	0.012	1.938 (1.157–3.248)
NLR, ≥10% decrease at 1st response	0.037	0.002	0.305 (0.144–0.643)	0.586		
PLR, ≥150	0.134			0.743		
LMR ≥3.0	0.551			0.532		

Abbreviations: AFP, alpha‐fetoprotein; ALBI, albumin‐bilirubin; BCLC, Barcelona clinic liver cancer; DCP, des‐gamma‐carboxy prothrombin; ECOG PS, European Cooperative Oncology Group performance status; LMR, lymphocyte to monocyte ratio; NLR, neutrophil to lymphocyte ratio; PLR, platelet to lymphocyte ratio.

### 3.4 Adverse events (AEs)

The safety profiles in terms of AEs are shown in Table 4. A total of eighty‐five (70.2%) patients experienced any grade of AEs. The most common AEs of any grade were hypertension (44.6%), followed by thrombocytopenia (37.2%), fatigue (36.4%), and AST elevation (34.7%). Grade 3 or 4 AEs occurred in 33 (27.3%) of the patients, and the most common were AST elevation (7.4%), hypertension (4.1%), and proteinuria (4.1%). Among four patients with gastrointestinal (GI) bleeding, two had duodenal ulcer bleeding and two had esophageal variceal bleeding. GI perforation occurred in three patients. Intracranial hemorrhage and pulmonary embolism were present in one patient each. Eight (6.6%) patients discontinued drugs due to AEs. All three patients with cessation of ATE exhibited liver function deterioration. Five patients discontinued BEV due to GI bleeding or perforation (*n* = 4) and intracranial hemorrhage (*n* = 1).

**TABLE 4:** Adverse events

	Any grade	G3‐4	G1‐2
Hypertension	54 (44.6)	5 (4.1)	49 (40.5)
Thrombocytopenia	45 (37.2)	4 (3.3)	41 (33.9)
Fatigue	44 (36.4)	0	44 (36.4)
AST elevation	42 (34.7)	9 (7.4)	33 (27.3)
Proteinuria	35 (28.9)	5 (4.1)	30 (24.7)
Anemia	30 (24.8)	1 (0.8)	29 (24.0)
ALT elevation	24 (19.8)	3 (2.5)	21 (17.4)
Blood bilirubin increase	24 (19.8)	2 (1.6)	22 (18.2)
Nausea	24 (19.8)	1 (0.8)	23 (19.0)
Anorexia	23 (19.0)	0	23 (19.0)
Neutropenia	14 (11.6)	3 (2.5)	11 (9.1)
Rash	13 (10.7)	2 (1.7)	11 (9.1)
Pruritus	13 (10.7)	3 (0.8)	10 (8.2)
Gastrointestinal bleeding	6 (5.0)	4 (3.3)	2 (1.7)
Vomiting	6 (5.0)	0	6 (5.0)
Diarrhea	6 (5.0)	0	6 (5.0)
Gastrointestinal perforation	3 (2.5)	3 (2.5)	0
Hypothyroidism	1 (0.8)	0	1 (0.8)
Intracranial hemorrhage	1 (0.8)	1 (0.8)	0
Pulmonary embolism	1 (0.8)	1 (0.8)	0

Abbreviations: ALT, alanine aminotransferase; AST, aspartate aminotransferase.

## 4 DISCUSSIONS

Through the present real‐world multicenter study, we showed that ATE/BEZ has acceptable efficacy to treat patients with advanced stage HCC. Both OS and PFS were generally compatible with prior reports.^3^, ^4^ As predictive biomarkers, a baseline high DCP level and NLR were correlated with poor PFS and OS. A decrease in the NLR at the first response was a favorable predictor for OS. Although early decreases of AFP and DCP levels were correlated with ORR, they did not have a significant effect on OS or PFS.

This study had several strengths. First, we investigated the real‐world clinical data on ATE+BEV regimen which has been recently positioned as the 1st‐line treatment for advanced HCC in the 2022 update of BCLC strategy.^5^ Second, we suggested two potential clinical biomarkers (DCP and NLR) for HCC. Because tissue biopsy is not mandatory for diagnosis of HCC, the discovery of predictive biomarkers through tumor tissue is difficult in HCC compared with other solid cancers. Therefore, the implications of clinical biomarkers in the era of molecular target agents and/or immune‐check point inhibitor may be crucial in HCC. Mainly, baseline DCP level in this study was significantly predictive for patient prognosis. Compared with AFP, which is nonspecific because it reflects regeneration of hepatocytes and often increases in benign conditions such as hepatitis or cirrhosis,^13^, ^14^ a high serum level of DCP is associated with more aggressive tumor behavior, such as a poor histologic grade of tumor differentiation, presence of intrahepatic metastasis, and presence of MVI.^15^, ^16^ In addition, high baseline NLR was an unfavorable predictive marker for ORR, PFS, and OS. As NLR stands for the neutrophil counts/lymphocyte counts where both components are easily available from the routine complete blood count,^17^ an elevated NLR suggests neutrophilia or lymphopenia and is generally regarded as a systemic inflammatory marker. Neutrophilia is associated with cancer‐promoting chronic inflammation; conversely, lymphopenia is associated with decreased lymphocyte‐mediated adaptive immunity. Indeed, significant associations of an elevated NLR with poor PFS or OS have been demonstrated in many kinds of malignancy, including HCC treated with various treatment modalities.^18^, ^19^, ^20^, ^21^ Moreover, a prognostic role of NLR in patients with HCC treated with immune‐checkpoint inhibitor has been also reported in the era of immunotherapy^22^, ^23^, ^24^ as well as in patients receiving molecular target agents. In a study by Nakano et al., baseline NLR as well as changes of NLR after one month of treatment was prognostic in patients with advanced HCC receiving molecular target agents.^25^ Lastly, it is noteworthy that a decrease in NLR from baseline, as well as baseline NLR value, was predictive of OS. Therefore, NLR, an easily accessible biomarker from clinical practice, is expected to serve as baseline and on‐treatment biomarker for patients receiving ATE+BEV. NLR may enable identification of patients who will benefit from ATE+BEV, facilitating the selection of those who need close monitoring or a switch to rescue therapy.

Regarding early tumor marker responses, early AFP reduction (*p* = 0.017) and DCP reduction (*p* = 0.032) were significantly associated with the ORR; however, their associations with survival outcomes were insignificant. This might be attributed to an insufficient follow‐up duration. Unlike prior reports that systemic agents with low ORRs had weak correlations with PFS and/or OS, recent systemic agents with high ORRs have strong correlations with survival outcomes. Therefore, additional researches on a feasible on‐treatment biomarker that shows correlations with both ORR and survival outcomes are needed. In the similar context, neither an early AFP nor DCP response was associated with OS. Therefore, further studies are required to identify early on‐treatment biomarkers predictive of survival outcomes.

This study had some drawbacks. First, because our study had a retrospective design with a relatively small sample size and insufficient follow‐up, bias might occur. In particular, since reimbursement for ATE+BEV treatment was limited in the Republic of Korea at that time, only patients who could afford to pay out‐of‐pocket were included in this study. Therefore, the subsequent well‐designed studies based upon the larger sample size and a longer follow‐up duration should be necessary in the near future. Second, we evaluated only clinical biomarkers. Because translational biomarkers such as angiopoietin‐2, VEGF‐A, and vascular cell adhesion molecule‐1, tumor mutational burden, and other gene signatures were not assessed,^26^ further studies using laboratory and histological samples are warranted to guide more information for treatment.

In conclusion, the combined regimen of ATE+BEV to treat patients with advanced stage HCC provided not only acceptable efficacy but also tolerable safety in the real‐world practice in the Republic of Korea, consistent with the reports from the IMbrave 150 trial. Baseline DCP and NLR, as well as early NLR decline, may serve as predictive biomarkers among HCC patients treated with ATE+BEV.

## AUTHOR CONTRIBUTIONS

Conception: Beom Kyung Kim, Young Eun Chon, and Hong Jae Chon; study design: Beom Kyung Kim, Jaekyung Cheon, Young Eun Chon, and Hong Jae Chon; participation in patient management and data collection: Beom Kyung Kim, Jaekyung Cheon, Hyeyeong Kim, Beodeul Kang, Yeonjung Ha, Do young Kim, Seong Gyu Hwang, Young Eun Chon, and Hong Jae Chon; contribution to the data acquisition, responsibility for writing the paper, and statistical analysis; Beom Kyung Kim, Jaekyung Cheon, Young Eun Chon, and Hong Jae Chon. All authors have reviewed the paper and approved the final version.

## CONFLICT OF INTEREST

HJ Chon has received honoraria from Eisai, Roche, Bayer, ONO, MSD, BMS, Celgene, Sanofi, Servier, AstraZeneca, Sillajen, Menarini, GreenCross Cell, Boryung Pharmaceuticals, Dong‐A ST, and has received research grants from Roche, Dong‐A ST, Boryung Pharmaceuticals. J Cheon received research grants from Bayer and honoraria from Eisai, Servier and Roche. The other authors have no potential conflicts of interest to disclose.

## Data Availability

n/s.
